# Sex and ABO Blood Differences in SARS-CoV-2 Infection Susceptibility

**DOI:** 10.1055/s-0043-1761202

**Published:** 2023-01-30

**Authors:** Mahmut Cerkez Ergoren, Gokce Akan, Emrah Guler, Gulten Tuncel, Damla Akovalı, Emine Unal Evren, Hakan Evren, Huseyin Kaya Suer, Tamer Sanlidag

**Affiliations:** 1 Department of Medical Genetics, Faculty of Medicine, Near East University, Nicosia, Cyprus; 2 DESAM Research Institute, Near East University, Nicosia, Cyprus; 3 Department of Nutrition and Dietetics, Faculty of Health Sciences, Near East University, Nicosia, Cyprus; 4 Blood Bank Unite, Near East University Hospital, Near East University, Nicosia, Cyprus; 5 Department of Infectious Diseases and Clinical Microbiology, Faculty of Medicine, University of Kyrenia, Kyrenia, Cyprus; 6 Department of Infectious Diseases and Clinical Microbiology, Faculty of Medicine, Near East University, Nicosia, Cyprus

**Keywords:** ABO blood group, Rh blood type, SARS-CoV-2, COVID-19, Turkish Cypriot

## Abstract

Data consisting of millions of cases cannot still explain the immunopathogenesis mechanism between severe acute respiratory syndrome coronavirus 2 (SARS-CoV-2) infection and host cell for ongoing coronavirus disease 2019 (COVID-19) pandemics. Epidemiological studies among different populations suggested different impacts of ABO and Rh antibodies on the COVID-19 susceptibility. Thus, the ABO blood group and the SARS-CoV-2 infection paradox remain unclear. Therefore, the present retrospective case–control study aimed to investigate the possible association between ABO blood groups and Rh blood types on SARS-CoV-2 infection in the Turkish Cypriot population. A total of 18,639 Turkish Cypriot subjects (297 SARS-CoV-2 COVID-19 patients and 18,342 healthy) were included in this study. Personal and clinical characteristics including age, gender, SARS-CoV-2 infection status, the ABO blood group and Rh blood types were evaluated and compared between two groups. As a result, ABO blood group was shown to be associated with a higher risk of SARS-CoV-2 infection as well as with male sex ( *p* = 0.018). There was no association between Rh blood type and COVID-19. Overall, this study is the first largest sample group study to show the distribution of ABO blood group and Rh blood types in the healthy Turkish Cypriot population. Based on the current evidence, there are insufficient data to guide public health policies regarding COVID-19 pathogenesis.

## Introduction

Diagnostic, preventive, and therapeutic studies on severe acute respiratory syndrome coronavirus 2 (SARS-CoV-2), which was reported as a pneumonia epidemic of unknown cause in December 2019 in Wuhan and was later named as new coronavirus disease 2019 (COVID-19) by the World Health Organization (WHO) in January 2020, are continuing intensively on a global scale. Even though vaccination against SARS-CoV-2 has significantly decreased hospitalization and mortality rates, emergence of new variants with higher transmission rates prevents the complete eradication of the disease.

Dry cough, shortness of breath, and high fever were reported as the most common symptoms of the COVID-19 infection, while some infected patients remained completely asymptomatic. 1 As the reason for this heterogeneity in symptoms associated with COVID-19 in infected patients remains mostly unclear, elucidating the risk factors underlying susceptibility of individuals to infection, mortality, and disease severity has been important in establishing preventive strategies and understanding the molecular mechanism underlying infection and spread. Studies have indicated several genetic variations that affect the susceptibility of individuals with the virus and the severity of disease. 2 A well-studied example is the angiotensin-converting enzyme 2 (ACE2) that encodes for the SARS-CoV-2 host receptor protein. The spike transmembrane glycoprotein (S) on SARS-CoV-2 surface helps the virus cohere to a host cell by attaching to the ACE2 cell-surface receptor. Several epidemiological studies revealed *ACE2* gene variations that alter the amino acid sequence of the receptor protein can affect interindividual variability and susceptibility to COVID-19 in populations. 3 4 5 Similarly, variations in transmembrane protease serine 2 ( *TMPRSS2* ) that is involved in priming SARS-CoV-2 by cleaving the S protein at the S1/S2 and S2 sites were shown to affect susceptibility of individuals to be infected with the virus by altering or initiating cell entry. 6 7

In addition to genetic variations, gender, age, chronic disease state, serum vitamin D levels, nutrition, and blood groups are among the factors that are investigated for COVID-19 susceptibility. 8 9 10 11 Previous studies have proposed that blood group antigens can act as molecular receptors and coreceptors for pathogens and facilitate intracellular uptake of microorganisms, parasites, and viruses, including SARS-CoV-2. 12 13 A study by Guillon et al showed human anti-A antibodies inhibited interaction between ACE2-dependent cellular adhesion and ACE2-expressing cells using a cellular model of adhesion, suggesting a mechanism by which ABO polymorphism affects susceptibility to SARS-CoV-2 infection and transmission. 14 Epidemiological studies in different populations proposed different effects of ABO and Rh antibodies in COVID-19 susceptibility. 15 16

In the light of these information, this study aims to investigate any significant difference between ABO blood groups and Rh blood types on SARS-CoV-2 patients compared with the general population in Northern Cyprus.

## Materials and Methods

### Case and Control Selection

The present retrospective case–control study included unrelated 18,639 Turkish Cypriot subjects. The ethnicity was defined as being born Turkish Cypriot couples for at least three generations. Personal and clinical characteristics including age, gender, SARS-CoV-2 infection status, ABO blood group, and Rh blood types were indicated. Total 297 subjects, who were diagnosed with COVID-19 based on SARS-CoV-2 real-time polymerase chain reaction (RT-PCR) approach (according to manufacturer guidelines of UNIPLEX SARS-CoV-2 RT-PCR Detection Kit, Nicosia, Northern Cyprus) between October 2021 and February 2022 at the Near East University Hospital COVID-19 PCR Diagnostic Laboratory, constituted the case group; 18,342 otherwise healthy, considering no evidence of SARS-CoV-2 RT-PCR positivity, individuals, who were already registered in the Blood Bank Unite, constituted the control group. Subjects who are younger than 18 years were excluded from both groups. This study was approved by the ethics committee of the university (application number: YDU/2021/87-1271) and performed with the ethical principles of the Declaration of Helsinki.

### Statistical Analyses

All data collected were organized in an excel database for Windows 10 (Microsoft Corporation, Redmond, Washington, United States) and analyzed in Statistical Package for Social Sciences 25.0 (IBM SPSS, Inc., Chicago, Illinois, United States).

The differences in the distribution of ABO blood group and Rh blood types among the studied groups were examined using chi-square tests for the categorical variables. Normal and nonnormal distributed quantitative variables were compared using the Student's *t* -test and Mann–Whitney's *U* test. The results were expressed as the mean ± standard deviation, or percentage, wherever appropriate.

The odds ratio (OR) test was applied to study the odds of ABO blood group and Rh blood types. ORs were reported with 95% confidence intervals (CIs). Statistical significance was considered when *p* < 0.05.

## Results

A total of 18,639 (297 COVID-19 patients and 18,342 controls) were included in the study. The mean age of the COVID-19 patients was 38.23 ± 19.57 and for the controls were 40.27 ± 18.04 years. The distributions of gender were 124 (41.8%) females and 173 (58.2%) males for COVID-19 cases and 7,319 (39.9%) females and 11,023 (60.1%) males for the controls. Statistically, the control and case groups were matched according to their age and sex.

The highest frequency was observed in the blood group A (42.8%), followed by O (34.8%), B (15.3%), and AB (7.1%). A total of 297 SARS-CoV-2-infected patients showed the similar distributions, where blood group A (44.4%), followed by O (29.0%), B (13.8%), and AB (12.8%) were reported from the highest to the lowest. Additionally, the distribution of the blood groups was statistically significant among the studied groups due to AB blood group ( *p* = 0.001, OR: 0.93, 95% CI: 0.83–1.03). The analysis showed that AB blood group was associated with a high risk of SARS-CoV-2 infection.

Furthermore, the distribution of Rh blood types in the control group was Rh (+) ( *n* = 16,541, 90.2%) and Rh (−) ( *n* = 1,801, 9.8%) and for the SARS-CoV-2-infected individuals were Rh (+) ( *n* = 274, 92.3%) and Rh (−) ( *n* = 23, 7.7%), respectively ( Table 1 ). There was no significant difference between the Rh blood types in the both studied groups ( *p* = 0.137, OR: 0.77, 95% CI: 0.50–1.18).

**Table 1:** Distribution of ABO blood group and Rh blood type among study groups

ABO blood group	COVID-19 patients *N* (%)	Controls *N* (%)	*p* -Value	OR (95% CI)
O	86 (29.0)	6,389 (34.8)	0.001 a	0.93 (0.83–1.03)
A	132 (44.4)	7,845 (42.8)		
AB	38 (12.8)	1,303 (7.1)		
B	41 (13.8)	2,805 (15.3)		
Rh blood type				
Rh (+)	274 (92.3)	16,541 (90.2)	0.137	0.77 (0.50–1.18)
Rh (−)	23 (7.7)	1,801 (9.8)		

Abbreviations: CI, confidence interval; COVID-19, coronavirus disease 2019; *N* , number of subjects; OR, odds ratio.

a Statistical significance.

There was a significant association between the distribution of ABO blood group and sex as well as Rh blood types within the case group ( *p* = 0.018, OR: 0.77, 95% CI: 0.60–0.98 and *p* = 0.186, OR: 0.52, 95% CI: 0.22–1.23, respectively) ( Table 2 ). The AB blood group was observed more prevalent in male COVID-19 patients than in female COVID-19 patients (17.3 and 6.5%, respectively). This result suggested that the AB blood group could be more prone to COVID-19 in male patients than the female patients.

**Table 2:** The distribution of ABO blood group and Rh blood types among sex in COVID-19 patients

ABO blood group	Female *N* (%)	Male *N* (%)	*p* -Value	OR (95% CI)
O	39 (31.5)	47 (27.2)	0.018 a	0.77 (0.60–0.98)
A	63 (50.8)	69 (39.9)		
AB	8 (6.5)	30 (17.3)		
B	14 (11.3)	27 (15.6)		
Rh blood type				
Rh (+)	111 (89.5)	163 (94.2)	0.186	0.52 (0.22–1.23)
Rh (−)	13 (10.5)	10 (5.8)		

Abbreviations: CI, confidence interval; COVID-19, coronavirus disease 2019; *N* , number of subjects; OR, odds ratio.

a Statistical significance.

## Discussion

Ongoing COVID-19 pandemics will be known as the longest among the last four pandemics on the earth resulting in 438,968,263 confirmed cases with 5,969,439 reported deaths. 17 Although it is controversial and still is not proven, there are studies showing that there is a relationship between blood types and contracting SARS-CoV-2 infection or the course of the disease. 18 It is critical to identify risk factors or risk groups to prevent wild spread infections and reduce mortality rates. Despite reported millions of cases, there is still a lack of information about demographic and clinical risk factors susceptible to SARS-CoV-2 infection. 19 Thus, the current study aimed to investigate the putative association between the SARS-CoV-2 infection and ABO blood groups as well as Rh blood types in the Turkish Cypriot population.

Rana et al (2021) showed that A or B blood groups were associated with a higher risk of having SARS-CoV-2 infection causing a COVID-19 than individuals with blood types O and AB as well as Rh (+) was found to be associated with the infection in Indian population. 20 Kerbage et al (2022) indicated that A Rh (+) subjects are more susceptible to SARS-CoV-2 infection; however, ABO blood groups lack any association with the severity of the disease. 21 Supporting the Indian study, Behera et al (2022) emphasized that blood group A was the most sensitive group considering a higher risk severity of the disease with admission to the intensive care unit. 19 In the present study, a total number of 18,639 (297 COVID-19 patients and 18,342 controls) individual's current data were investigated including SARS-CoV-2 RT-PCR results, sex, age, ABO blood groups, and Rh blood types. The most common Mediterranean blood groups, A and O, were found to have the two highest blood groups both in control and patient groups in the Turkish Cypriot population. The analysis showed that AB blood group was associated with a high risk of SARS-CoV-2 infection ( *p* = 0.001, OR: 0.93, 95% CI: 0.83–1.03). On the other hand, no significant association was found in the Rh blood types ( *p* = 0.137, OR: 0.77, 95% CI: 0.50–1.18).

In the literature, there was no study showing the relationship between ABO blood group and Rh blood types and SARS-CoV-2 infection in the Turkish Cypriot population. However, Solmaz and Araç (2021) showed the protective effect of O blood group in the Turkish population in 1,667 patients, whereas the A blood group might be associated with the COVID-19 susceptibility. 22 In addition, Dal et al (2021) indicated that ABO and Rh blood groups did not have any effect on hospitalization, hospital and intensive care stay rates, mechanical ventilation support, and mortality rate in the same population. 23

On the other hand, Latz et al (2020) showed that patients with B and AB blood groups and patients with Rh (+) blood type had a higher probability of SARS-CoV-2 RT-PCR positive results. 24 In a meta-analysis study covering 715 articles in the literature emphasized the need for more sensitive and high-quality research evidence, while showing A and B blood groups might be risk factors for COVID-19. 25

Another valuable result in the study was obtained that male individuals were more prone to SARS-CoV-2 positivity among Turkish Cypriots ( *p* = 0.018, OR: 0.77, 95% CI: 0.60–0.98). This finding was consistent with the current literature 26 27 as well as deaths caused by the COVID-19 were 2.4-fold in men. 28

The paradox of the relationship between ABO blood group and SARS-CoV-2 infection can be explained by several hypotheses. Anti-A and/or anti-B antibodies in blood group O individuals can bind to compatible antigens on the viral envelope and thus contribute to neutralization, resulting in preventive target cell infection, 29 and the situation may be the opposite in individuals with AB blood group. No doubt, more prospective studies are needed to confirm the proposed hypothesis. The limitation of this study could be considered the low number of individuals with B and AB blood groups as well as Rh (−) blood types in the Mediterranean region. In addition, SARS-CoV-2 RT-PCR positivity would be evaluated with the disease severity.

## Conclusion

Overall, this study is the first largest sample group study to show the distribution of ABO blood group and Rh blood types in the healthy Turkish Cypriot population. AB blood groups was shown to be associated with a higher risk of SARS-CoV-2 infection with male sex in this study. Unfortunately, based on the current knowledge and evidence, there are no sufficient data to guide policy on the COVID-19 immunopathogenesis.
